# Enabling beam-scanning antenna technologies for terahertz wireless systems: A review

**DOI:** 10.1016/j.fmre.2024.10.003

**Published:** 2024-11-12

**Authors:** Dongze Zheng, Geng-Bo Wu, Zhi Hao Jiang, Wei Hong, Chi Hou Chan, Ke Wu

**Affiliations:** a School of Information Science and Engineering, State Key Laboratory of Millimeter Waves, Southeast University, Nanjing 210096, China; b Department of Electrical Engineering, State Key Laboratory of Terahertz and Millimeter Waves, City University of Hong Kong, Hong Kong 999077, China; c Department of Electrical Engineering, Poly-Grames Research Center, Polytechnique Montréal, Montréal, QC H3T 1J4, Canada

**Keywords:** Beam-scanning, Frequency scanning, Leaky-wave antenna, Mechanical scanning, Microwave photonics, Phased array, Reconfigurable metasurface, Terahertz

## Abstract

Due to the exponentially growing global mobile data of wireless communications evolving from 5 G to 6 G in recent years, research activities of leveraging terahertz (THz) waves to obtain larger channel capacities have shown an ever-increasing pace and reached an unprecedented height than before. Historically, the past few decades have already witnessed much progress in THz generation and detection technologies, which have been recognized for a long time as the bottleneck preventing the THz waves from being tamed by human beings. However, the importance of developing advanced components such as antennas, transmission lines, filters, power amplimers, etc., which constitute the basic building blocks of a THz wireless system, should not be overlooked for the sake of exploiting the THz spectra for future advanced wireless communications, sensing and imaging applications. While producing a scannable highly-directive antenna beam proves to be indispensable in the period of microwaves, the significance of such functionality is more critical in the THz era, considering that THz waves have more intractable challenges such as the severity of free-space propagation losses, the susceptibility to atmospheric environments, and the unavailability of efficient signal sources. This article is structured under this background, which is dedicated to reviewing several enabling beam-scanning antenna concepts, structures, and architectures that have been developed for THz wireless systems. Specifically, we divide these THz beam-scanning solutions into four basic groups based on different mechanisms, i.e., mechanical motion, phased array, frequency beam-scanning, and reconfigurable metasurfaces.

## 1 Introduction

The terahertz (THz) wave is generally defined as the electromagnetic wave whose spectrum lies between 0.1 and 10 THz, and the first use of the term “terahertz” to describe such a spectral range can date back to the 1970s [1]. This spectral portion is sandwiched between and partially overlapped with the microwaves (electronics) and the infrared lights (photonics). Due to this, the THz wave inherits some technical characteristics from both microwaves and optical lights, which makes it capable of combining benefits from the two neighbors and presents uniqueness in a wide variety of applications such as communication, sensing, and imaging [[2], [3], [4], [5], [6]]. Moreover, the THz wave is in the molecular spectrum, making it capable of effectively modulating ion channels and activating nerves nonthermally and resonantly interacting with key molecules [7]. While microwave and optical regions have been well developed since the last century, the THz-related technologies have not kept the same pace with them, largely caused by the difficulties in generating and detecting THz waves [8,9]. This is also why the term “THz gap” was coined to describe the dilemma of using this special electromagnetic spectrum. Although pursuing effective generation and detection technologies is undoubtedly critical to enabling THz waves for wireless applications, developing some other THz functional components/circuits/devices is also indispensable. For example, research activities on passive and active THz components such as antennas, filters, transmission lines, power amplifiers, etc., should also be conducted to facilitate a workable THz wireless system.

While the serious propagation losses of THz waves cannot be avoided in free-space wireless transmission, it seems that we have no choices but to wisely resort to using high-gain antennas to realize a reasonable propagation distance considering the current technical limitations in enhancing the transmitter's power and receiver's sensitivity, as suggested by the famous Friis transmission formula [10]. This strategy was previously adopted when microwaves/millimeter-waves were put into practical use such as 5 G communications [11] and automotive radars [12]. However, the necessity of deploying high-gain antennas in THz wireless systems is more critical than that in microwaves, largely because THz waves suffer from more severe challenges like free-space path loss (due to the higher frequency), atmospheric attenuation (large absorption losses caused by water and oxygen molecules), and the powerful sources (lack of efficient power amplifiers or photodiodes) [8,9]. While there is a natural contradiction between the high gain and wide beam coverage, making the high-gain beam scannable in some fashions (i.e., a time-shared or concurrent manner) is seemingly the only solution to simultaneously satisfy the requirements of both the high-gain beam and the targeted spatial coverage or field-of-view.

The development of science and technology, despite of the subject or discipline, is generally gradual, which usually implies that past research activities/experiences can be good references to indicate the directions we can follow when facing new tasks or challenges. This fact is especially true when retrospectively observing the exploitation processes of electromagnetic spectra that increase from microwaves to THz waves. For example, when talking about the ways to generate high-frequency signals, using frequency-mixing or -multiplication is a typical method in the time of microwaves. This has also been adapted to generate THz waves and is classified as the full-electronics method [13]. Contrastively, microwave/millimeter-wave signals can be generated using the photo-mixing mechanism—a classical way belonging to the realm of microwave photonics or optoelectronics [14], which also become one of the mainstream approaches in generating THz waves nowadays [15]. Following this line of thinking and particularly focusing on the THz beam-scanning technologies [16,17], perhaps the first idea that comes to one's mind is to guess whether the existing beam-scanning solutions in the microwave region can be translated directly to the THz domain. We will confirm such a development pattern of beam-scanning technologies in THz waves by reviewing the previous efforts made on this topic in the past few decades, as will be particularly reviewed in this article. It will be shown that the beam-scanning approaches that are usually found in microwave regions, such the mechanical motion, phase arrays, frequency scanning, and reconfigurable metasurfaces, still constitute the main enablers in routing THz waves in the free space, as will be detailed in 2, 3, 4, 5, respectively. Notably, most of the photonics-assisted microwave beam-scanning methods such as using thermo-optical phase shifters, optical delay lines, and dispersive fiber-optic prisms are also applicable to the THz region [[14], [15], [16], [17]] with proper modifications; this will be detailed in Section 3. Conclusions and general remarks about those THz beam-scanning technologies are also drawn to end this review article, as can be found in Section 6.

## 2 Mechanical motion

Mechanically moving the part or whole of an antenna system (e.g., reflector and lens antennas) is the most representative way to obtain a scannable beam. Historically, this kind of beam-scanning was the dominant way in the early stage of the last century when the phased array technique was not mature due to the underdevelopment of phase shifters, T/R modules, and semiconductor technologies [18]. It still plays a significant role in nowadays wireless world such as being deployed in some microwave radar and imaging application scenarios while interestingly constituting one of the mainstream beam-scanning techniques in the THz band as well. Considering that mechanical movements such as translation or rotation are usually realized by using electromotors or servosystems in microwaves [19], these movement actions should be more sophisticated in THz waves because of the much shorter wavelength and smaller structural footprint than the counterparts of microwaves. To this end, advanced mechanical devices such as electromagnetic/piezoelectric actuators [[20], [21], [22], [23]] and microelectromechanical system (MEMS)-related techniques [[24], [25], [26]] should be deployed in THz systems to produce a delicate control of the antenna beam. Recall that mechanical beam-scanning schemes in microwaves can be generally grouped into two scales: (i) the macroscopical scale, such as moving the feeding horn [27,28] or the lens/reflector [29,30], and (ii) the microscopical scale, such as rotating the metasurface's elements [31,32]. The underlying implementation principles and multiscale classification criteria of microwave beam-scanning using mechanical motion can be properly transferred to the THz band, as discussed below.

### 2.1 Macroscopic scale

**Fig. 1:**
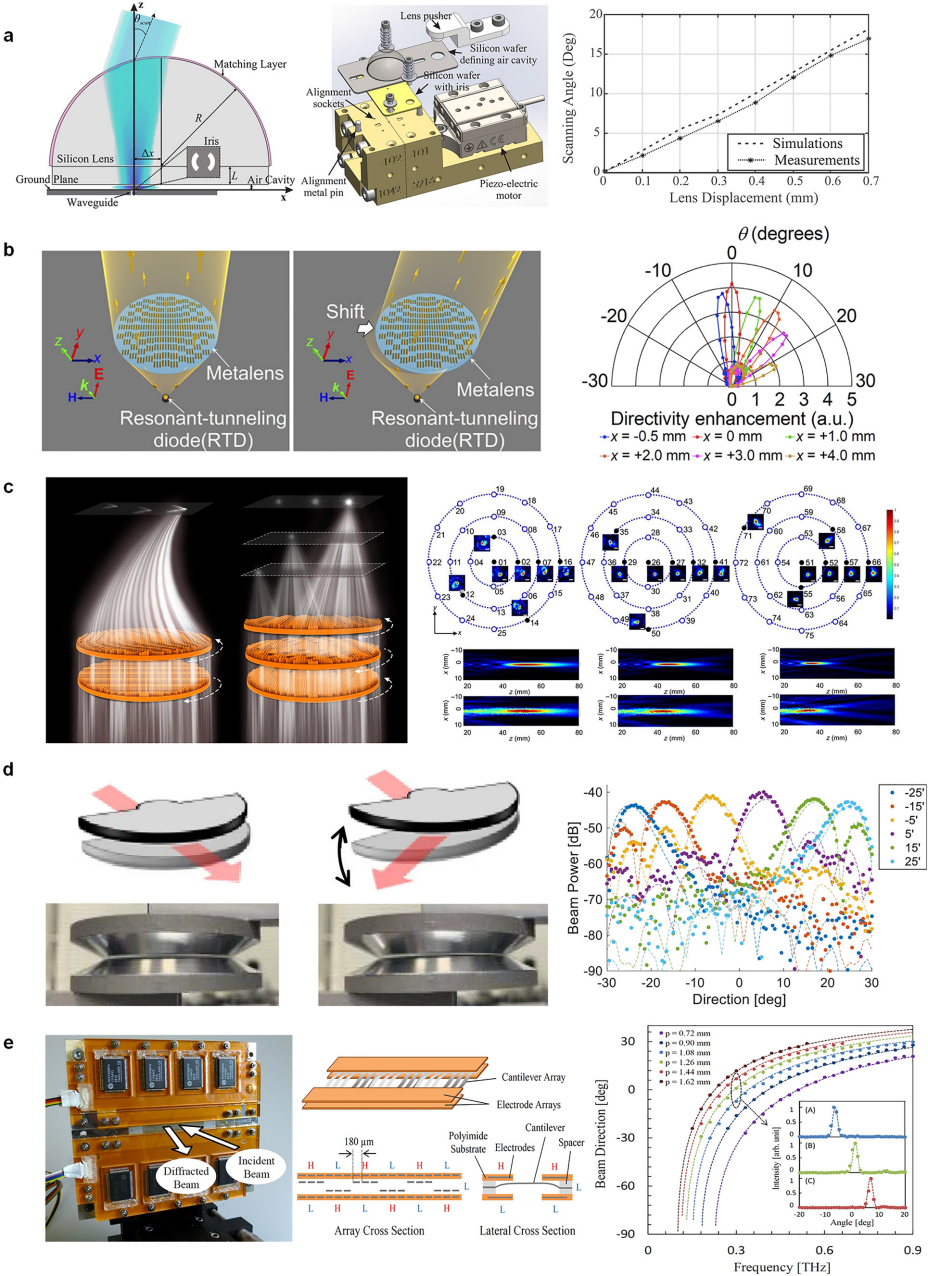
**Mechanical beam-scanning in THz waves**. (a) Movable silicon lens [21]. (b) Movable metalens/metasurface [33]. (c) In-plane rotation of triple metalenses for 3-D focus beam steering [34]. (d) Deformed PPWG lens with tiled bottom plate [41]. (e) Movable cantilevers that result in a period-variable diffraction grating [44]. Note that (a), (b), (c), and (d) belong to the macroscale-type mechanical beam-scanning, whereas (e) represents the counterpart of the microscale type.

Fig. 1a illustrates a typical example that uses a movable silicon lens and fixed feeding structure (i.e., leaky-wave cavity) to realize a scannable THz beam [21]. The hyper-hemispherical silicon lens is attached to a piezoelectrical actuator. The relative position of the lens with respect to the center of the leaky-wave cavity, or the beam direction, can be finely altered by controlling the piezoelectrical actuator with the supplied voltage. It is demonstrated that a larger displacement of the silicon lens can provide a beam angle that deviates more from the broadside direction. Sharing the similar principle of changing the relative position of the lens and feeding structure, a metalens antenna illuminated by a resonant-tunneling diode (RTD) was proposed in [33], where the entire body of the metalens is made horizontally moveable to deflect the beam, as shown in Fig. 1b. Contrastively, the in-plane rotation of lenses or metasurfaces is another approach to implementing beam scanning [34] as shown in Fig. 1c where the focused beam can be steered in 3-D space by in-plane rotating three 3-D printed metalenses. Following this mechanical mechanism, 2-D Airy beam scanning [34], 2-D Bessel beam scanning [35], and reconfigurable OAM modes [36] were also demonstrated. Notably, instead of moving the bodies of lenses/metasurfaces like the examples illustrated above, one can also physically change the location of feeding structures/ports to steer the beam [[37], [38], [39], [40]], which is quite understandable.

Dynamically deforming the main bodies of lenses/reflectors in some fashions is also a feasible way to establish a scannable beam in THz. Fig. 1d illustrates an example of this approach that depends on using a tilted metal plate of a TE_1_-mode-based parallel-plate waveguide (PPWG) lens [41]. While the TE_1_ mode is dispersive and has a cutoff frequency related to the distance between the two metal plates, the equivalent refraction index or phase velocity of the TE_1_-mode PPWG can be tailored by spatially changing the plate distance. In this sense, a tilted bottom plate can produce a gradual spatial variation of the plate distance and thus the equivalent refraction index, paving the way for deflecting the propagation direction of the electromagnetic waves inside the PPWG [42]. It was demonstrated that even a small tilt angle of the metal plate can produce a large deflection angle of the radiation beam. While the work using structural deformation in Fig. 1d is related to a guided-wave-type lens, a spatial-wave-illuminated deformed lens (or transmissive metasurface) made up of liquid crystal (LC) elastomers was proposed in [43]. In this work, the infrared light or thermal regulation mechanism is used to deform the lens/metasurface to generate a scannable beam.

### 2.2 Microscopic scale—element-level mechanical movement

Compared to imposing macroscopic motion or deformation on the lens/reflectors or feeding structures to obtain the desired THz beam-scanning, performing mechanical movement microscopically, or more specifically at the element level, also works for the same purpose. This beam-scanning mechanism can be exemplified by the work shown in Fig. 1e, where a programmable THz diffraction grating consisting of an array of electrostatically actuated metallic cantilevers was proposed [44]. Specifically, each cantilever can be moved vertically by applying a local electrostatic force that results from a bias voltage, upon which the spatial pattern of the cantilever array can be freely manipulated and so does the period. While the cantilevers’ period is a critical parameter that determines the beam direction of gratings, a scannable beam at a fixed frequency can be realized by adjusting the periodicity. Interestingly, different from using spatial-wave illumination like the case of Fig. 1e and the one proposed in [45], guided-wave-fed gratings presenting fixed-frequency mechanical beam-scanning behaviors can also be made following the similar period-variable principle [24,25]. It should be noted that compared to making efforts on the element-level mechanical movement in microwaves [19,31,32], it is more challenging to involve a similar action in the THz band because of the extremely small footprint of elements at such a high frequency. Fortunately, the advancement of MEMS technologies can enable the desired mechanical control at such a microscale level [26].

## 3 Phased beam-scanning

Phased array antennas generally consist of a series of discrete antenna elements. When properly phased, the radiated fields of these antenna elements will coherently interfere in the free space to form a directional beam, upon which a desired beam scanning can be realized by electrically changing the input phase without the need to mechanically move the array or feeding structures. Nevertheless, it should be noted that the phased beam-scanning techniques are generally associated with the way how to generate and/or distribute signals. This remark can be justified in the era of microwaves, at which time the perspective of performing phased beam scanning can be classified into full-electronics [11] and microwave photonics (or simply photonics) [14]. While THz waves can be similarly generated following the manners of generating microwaves, i.e., using the electrical up-conversion or photonic down-conversion (photomixing) of frequency [15], it is thus necessary to separately discuss THz phased beam-scanning techniques from such two perspectives, i.e., full-electronics and photonics.

### 3.1 Full-Electronics perspective

(a) Integrated Circuit (IC)-Based Phased Array

**Fig. 2:**
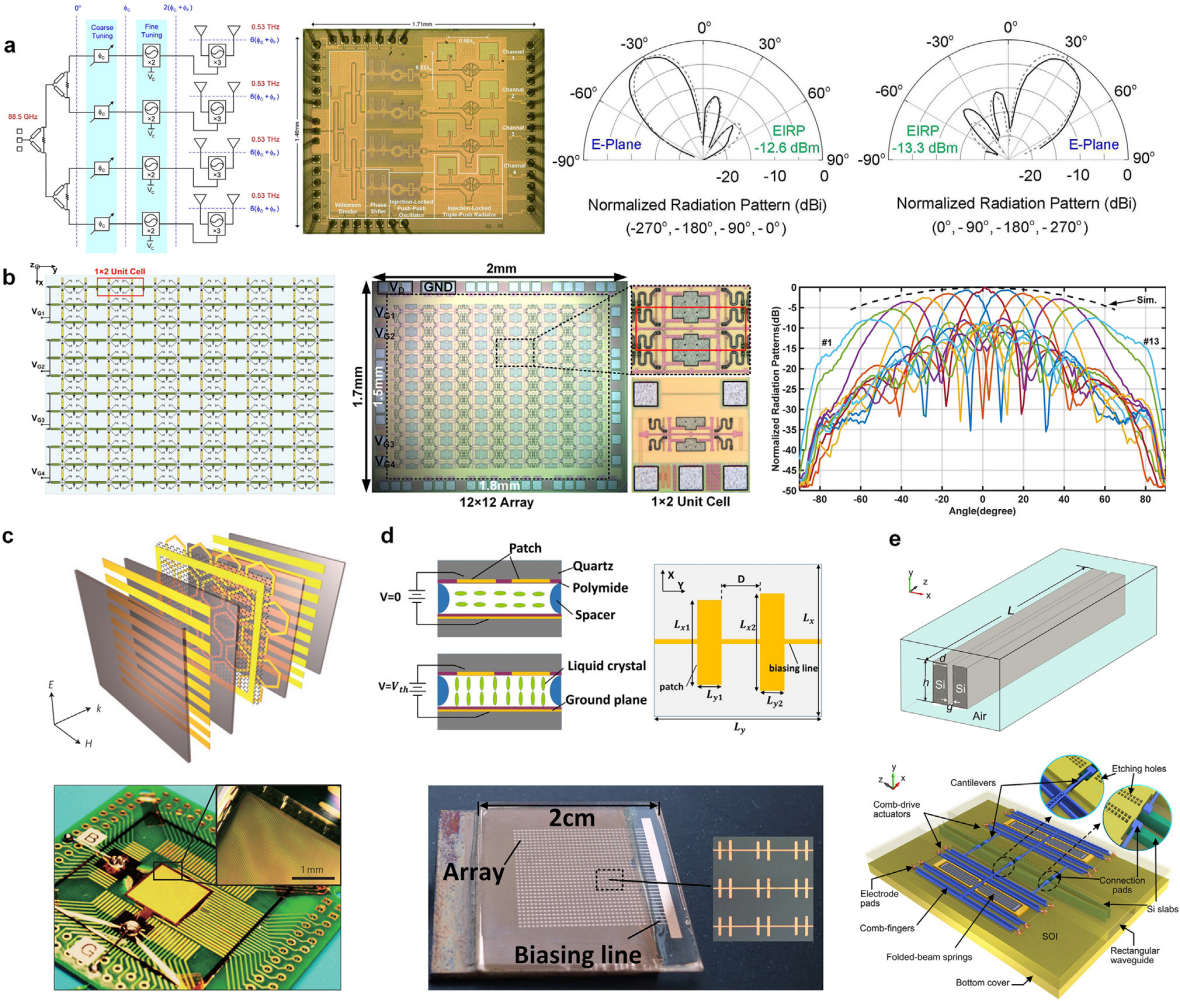
**THz phased beam scanning from the perspective of full-electronics**. (a) Subharmonic injection-locked phase array with phase controlling in the microwave band before up-conversion to the THz band [48]. (b) Coupled oscillated antenna array for direct phase control at THz frequencies [52]. (c) Graphene-based gate-controlled active metamaterials [55]. (d) LC-based THz phase shifter with over 360° phase tuning range [58]. (e) MEMS-based waveguide-integrated phase shifter [61].

Since THz waves can be generated through frequency up-conversion or multiplication from microwaves/millimeter waves, the phase shifting can be first implemented in the lower frequency band before upconverting signals to the THz band [[46], [47], [48], [49]]. For example, Yang *et al*. divide the W-band signal (90–105 GHz) into eight paths using a Wilkinson splitter, and then use phase shifters and amplifiers for phase and amplitude control before upconverting signals to the 360–420 GHz range through ×4 quadruplers [47]. This eight-element phased array achieves a 1-D beam scan range of ±35° based on 45-nm CMOS silicon on insulator technology. Notably, this architecture offers advantages such as reduced insertion loss in the feeding network and higher power amplifier gain. Due to the ×4 multiplication factor, the required phase shifting range at the lower frequency band is reduced to 90°, a quarter of the direct phase shifting in the THz band. However, any phase errors presented in the low-frequency band are multiplied in the THz band and thus may compromise practical beam scanning performance. To address this issue, one promising strategy is to use injection-locked oscillators (ILOs) [48] instead of traditional frequency multiplier chains. As shown in Fig. 2a, a 2-bit phase shifter enables coarse phase tuning at 88.5 GHz, while the first subharmonic ILO corrects the phase error by adjusting its free-oscillation frequency. Note that a second subharmonic ILO is cascaded to the first one to mitigate power variations while enabling THz wave generation. The injection-locked 1 × 4-element phased array can achieve E-plane beam scanning of 60° at 0.53 THz [48]. Following the same mechanism of performing phase shifting before the frequency up-conversion, an intermediate frequency (IF) beamforming architecture featuring 5-bit phase and 4-bit amplitude control at 9–14 GHz is adopted in an eight-element phased array transmitter operating at 140 GHz [50]. This phased array can achieve ±30° E-plane beam scanning and support a transmission data rate of 16 Gbps under the use of 16QAM and 64QAM waveforms.

The coupled oscillator topology stands out as a promising solution to realize the phase tuning and the relevant beam-scanning capability at the oscillator level [[51], [52], [53], [54]]. In this technique, adjacent oscillators are electrically interconnected and mutually synchronized, and the required phase adjustment is realized by individually manipulating the bias voltages of oscillators. A 12×12 coupled oscillator array that utilizes out-of-phase coupling between elements to differentially feed the patch antennas was proposed in [52], as shown in Fig. 2b. This 144-element phased array can achieve a beam scanning from −45° to 45° in the H-plane, with 9.1 dBm radiated power and a 30.8 dBm lensless effective isotropic-radiated power (EIRP) realized at 0.675 GHz. Note that the required phase-shifting process using coupled oscillators can also be implemented before the frequency is up-converted into the THz band [53].

(b) Phase Shifters Using Tunable Materials/Components

Another method for realizing the required phase-shifting mechanism and thus beam-scanning of phased arrays at THz is through incorporating tunable materials such as MEMS, LC, graphene, and vanadium dioxide (VO_2_) into the process of wave guidance/transmission. This subsection primarily focuses on discussing THz phase shifters based on tunable materials, while exploiting these phase shifters for THz beam-scanning arrays or metasurfaces will be discussed later in Section 5. One notable advancement is the development of a gate-controllable graphene-based metamaterial (see Fig. 2c) that enhances light-matter interaction, enabling a phase shift of 32.2° along with amplitude modulation of 47% for the transmitted waves [55]. In another design, a ring-dumbbell composite resonator integrated with VO_2_ material achieves a transmission phase range of 138° at 0.6 THz [56]. This enhancement in phase range is realized by leveraging the coupled dipole resonance and capacitive-inductance resonance of the meta-atom structure. In [57], a checkboard-like planar metamaterial or metasurface demonstrates impressive reconfigurable capabilities by transforming to its Babinet-inverted structure through the manipulation of the metal-insulator states of VO_2_. This transformation enables a switchable phase shift of 90° at 0.47 THz, showcasing the dynamic and versatile nature of VO_2_-based metamaterials in achieving precise phase control.

Apart from using exotic materials like graphene and VO_2_, we can also exploit LCs for developing phase-shifting devices [[58], [59], [60]]. LCs exhibit strong birefringence properties, and the orientation of LC molecules can be altered by applying an external electric field or bias voltage, upon which an adjustable refractive index can be obtained for the LCs. In [58], a reflective phase shifter employing two parallel quartz plates enclosing nematic LC achieves an impressive phase range of 362.6° at 357 GHz, as shown in Fig. 2d. While the work [58] utilizes two parallel unequal dipole metallic patterns as electrodes, Indium Tin Oxide (ITO) was utilized as transparent electrodes in a transmissive LC phase shifter as proposed in [59], which can provide a phase shift of > 90° and a transmittance exceeding 78% at 1 THz. Moreover, by actively switching the LC in both orientations using a grating electrode design, an LC-based phase shifter achieved a controllable phase shift range of 35° at 2 THz, with a switching time ranging from 10 to 470 milliseconds [60]. It is interesting to mention that LC-based phase shifters can be further expanded using the spatial feeding mechanism to form beam-steering reflectarrays, transmitarrays, and metasurfaces, as will be discussed in Section 5.

The MEMS-based THz phase shifters proposed in [[61], [62], [63], [64]] share a similar operation mechanism, which is simply based on tuning the propagation constant or equivalent refractive index of relevant THz transmission lines by partially changing their geometric structures with the help of sophisticated MEMS devices. For instance, the equivalent refractive index of a slot waveguide can be dynamically tuned over a broad frequency range by altering the distance between two parallel high-resistivity silicon slabs using comb-drive actuators, as shown in Fig. 2e. This approach has demonstrated a substantial phase range of 550° with a maximum insertion loss of 1.87 dB at 0.33 THz [61]. In another implementation, a series of MEMS-based waveguide stubs are positioned on the roof of a rectangular hollow waveguide to introduce controllable perturbations on the propagating TE_10_ mode, leading to a transmission phase shift of 20° within the frequency range of 0.5 to 0.55 THz [62]. In [63], a phase range of 145° is achieved, by employing a MEMS-based actuator to control the position of the perforated silicon slab that is inserted into a rectangular waveguide.

### 3.2 Photonics perspective

(a) Guided-Wave-Based Optically Controlled Phased Array

**Fig. 3:**
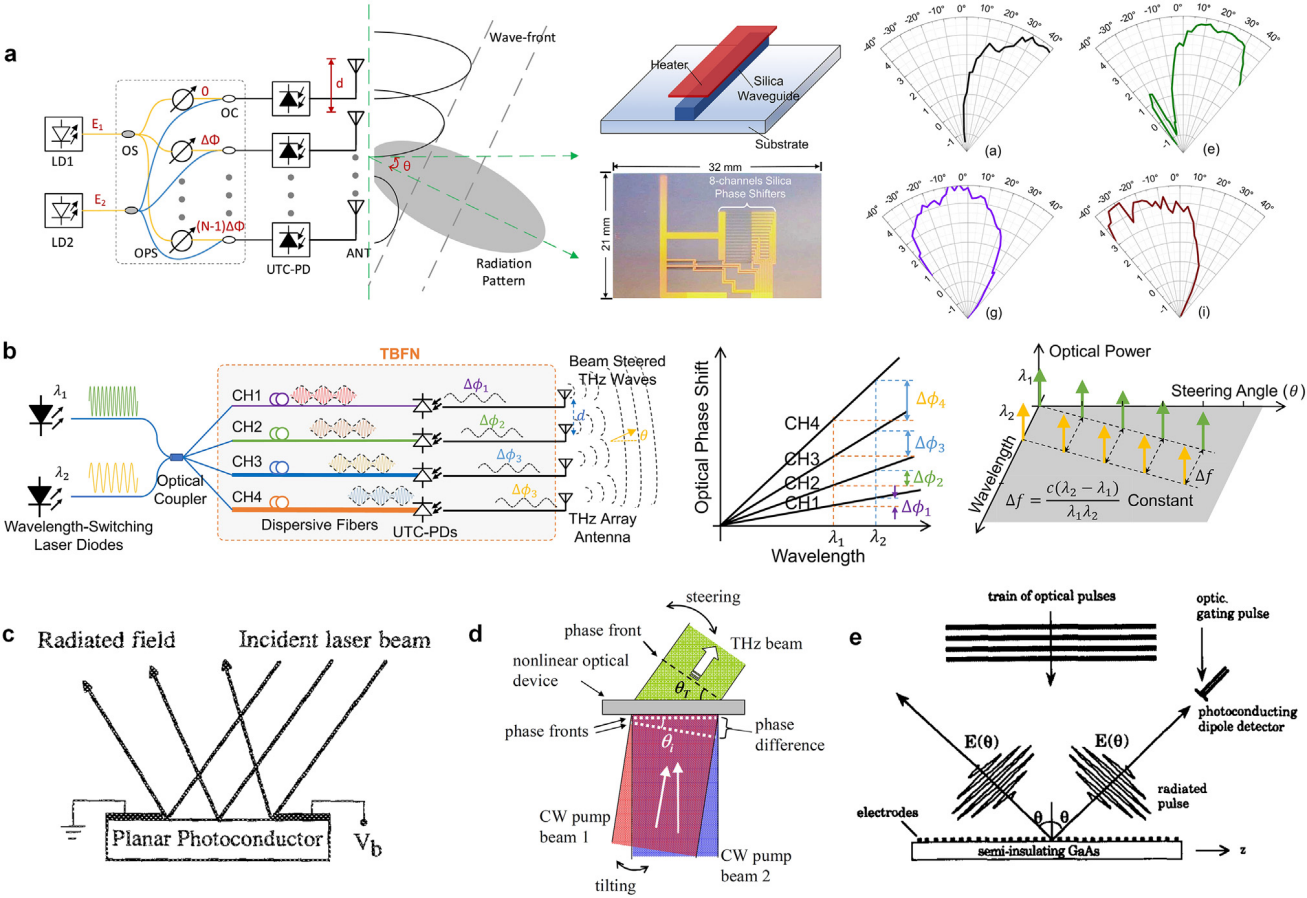
**THz phased arrays beam-scanning based on photonic technologies**. (a) Using photomixing together with thermo-optic phase shifters [65]. (b) Using photomixing together with the fiber chromatic dispersion [70]. (c) Using the specular reflection of PCAs together with changing the angle of the incident optical beam [76]. (d) Using photomixing together with tilting the angle of one optical pumping beam [77]. (e) Using a PCA array together with changing the spatial period of applied voltages [78].

The field of microwave photonics [14,15] indicates that performing optical heterodyne or photomixing of two lightwaves with the help of photodiodes can produce an output photocurrent that has a frequency equal to the difference frequency of the two lightwaves. Theoretically speaking, the frequency of the photocurrent can be arbitrarily selected from microwave to THz, depending on the availabilities of optical sources and the bandwidth of the photodiodes. While the phase of the generated photocurrent is the phase difference between the input two lightwaves, the phase shifting required for THz beam-scanning can be conducted by manipulating the phase of each of the two lightwaves before the photomixing process and in the optical domain [[65], [66], [67], [68], [69], [70]]. For example, two optical laser diodes working at 1550 nm and 1552.4 nm were used to generate a 300-GHz wave [65], as shown in Fig. 3a. In this work, silica-waveguide-based feeding networks are used to distribute the lightwaves to eight channels, each comprising a uni-traveling-carrier photodiode (UTC-PD) for photomixing and a slot antenna array for THz wave radiation in the free space. Thermo-optical phase shifters are incorporated into one of the distribution networks to impose a phase shift on one of the lightwaves before the UTC-PD. Each pair of the lightwaves is combined and then fed into the UTC-PD to generate THz waves with a spatial gradient phase distribution along the antenna elements. Note that apart from using optical phase shifters, leveraging the inherent chromatic dispersion of optical fibers represents another simple and effective method for implementing THz beam scanning [[68], [69], [70]]. Fig. 3b illustrates an example in this aspect [70], in which four optical fibers with specially engineered dispersion characteristics are deployed to generate a gradient phase difference of the two lightwaves along the UTC-PD antenna array. Since the phase shift is frequency-dependent, tuning the frequencies of the two lightwaves while maintaining an identical THz frequency difference is necessary to enable the required beam scanning of the THz waves. Interestingly, the mechanism of using fiber dispersion for beam-scanning can date back to the work of [71], which was used to optically control the beam direction of a microwave phased array with true-time delay performances. As a side note, the approach of using tunable laser sources or fiber Bragg grating prisms [[72], [73], [74], [75]], which was initially proposed for microwave phase arrays, can also be adapted to THz beam-scanning in principle if an additional laser source/optical carrier is used to trigger the photomixing process occurring at the photodiodes.

(b) Free-Space Optical Pumping.

While previously discussed optically controlled THz bean-scanning architectures rely on optical fiber or waveguiding structures for power transmission and distribution, the adoption of spatial optics or free-space optical pumping represents another effective method [[76], [77], [78], [79], [80]]. For example, considering that the photoconductive antennas (PCAs) under optical pulse illumination can generate a THz pulse that has a direction presenting a specular relation with that of the pumping optical pulse, the beam of the THs pulse can be thus steered by varying the incident angle of the optical pulse [76], as shown in Fig. 3c. This beam-steering mechanism resembles the mechanical motion solution introduced in Section 2, with the difference being the movement of the optical source rather than the THz source. Alternatively, the optical phase difference can be achieved by adopting two spatially dispersed optical pumping beams with different incident angles to illuminate nonlinear optical materials or photodiode arrays [77], as shown in Fig 3d. By tilting one of the incident optical pump beams by an angle of 0.155°, the phase gradient at the down-converted THz frequency changes significantly, enabling a THz beam scanning range of 29°. Note that only a small tilting angle can result in a large beam-scanning range, which is simply because the phase distribution for THz waves is formed by the spatial path difference at the optical frequency.

While the beam-scanning presented in [76,77] is somewhat related to a mechanical way, an electrical control enables higher-speed and more accurate beam scanning. As shown in Fig. 3e, a PCA array with a spatially sinusoidal bias distribution can be used to control the aperture excitation amplitude distribution [78]. Analogous to a diffraction grating that will be described later in Section 4, the −1st and +1st order space harmonics of the PCA array radiate into free space, and their beam directions are determined by the spatial period of the applied sinusoidal bias (in fact, the grating-related works in [44,45] and the work in [78] share the similar period-changing-based principle to enable beam-scanning, but they are differentiated by how the grating period is changed). Consequently, the radiated THz beam angle of the PCA array can be electrically steered over 40° with a stationary optical illumination. One limitation of ref. [78] is its dual-beam radiation since a single beam is generally required for practical use.

## 4 Frequency beam-scanning

Compared to the phased array technique that uses electrical or optical phase-shifting devices to realize the required beam-scanning, the frequency-enabled beam-scanning mechanism has general advantages of lower cost and lower system complexity because the beam can be scanned by simply changing the carrier frequency of signals [[81], [82], [83]]. While the phase-shifting devices in the THz band are either lossy or difficult to implement, the frequency beam-scanning technique can provide much practical significance in this case, especially being applied in THz sensing and imaging applications provided that the narrowband nature towards a certain direction has been well considered beforehand [[4], [5], [6]]. This section will discuss two basic groups of radiating structures for generating a frequency-scanned THz beam, i.e., the leaky-wave antennas (LWAs) [[84], [85], [86], [87], [88], [89], [90], [91], [92], [93], [94], [95], [96], [97], [98], [99], [100], [101], [102], [103], [104], [105]] and diffraction gratings [[106], [107], [108], [109], [110], [111], [112], [113], [114], [115], [116], [117], [118], [119], [120], [121]], which are characterized by guided-wave and spatial-wave excitation/illumination, respectively.

### 4.1 Leaky-wave antennas—the type using guided-wave excitation

**Fig. 4:**
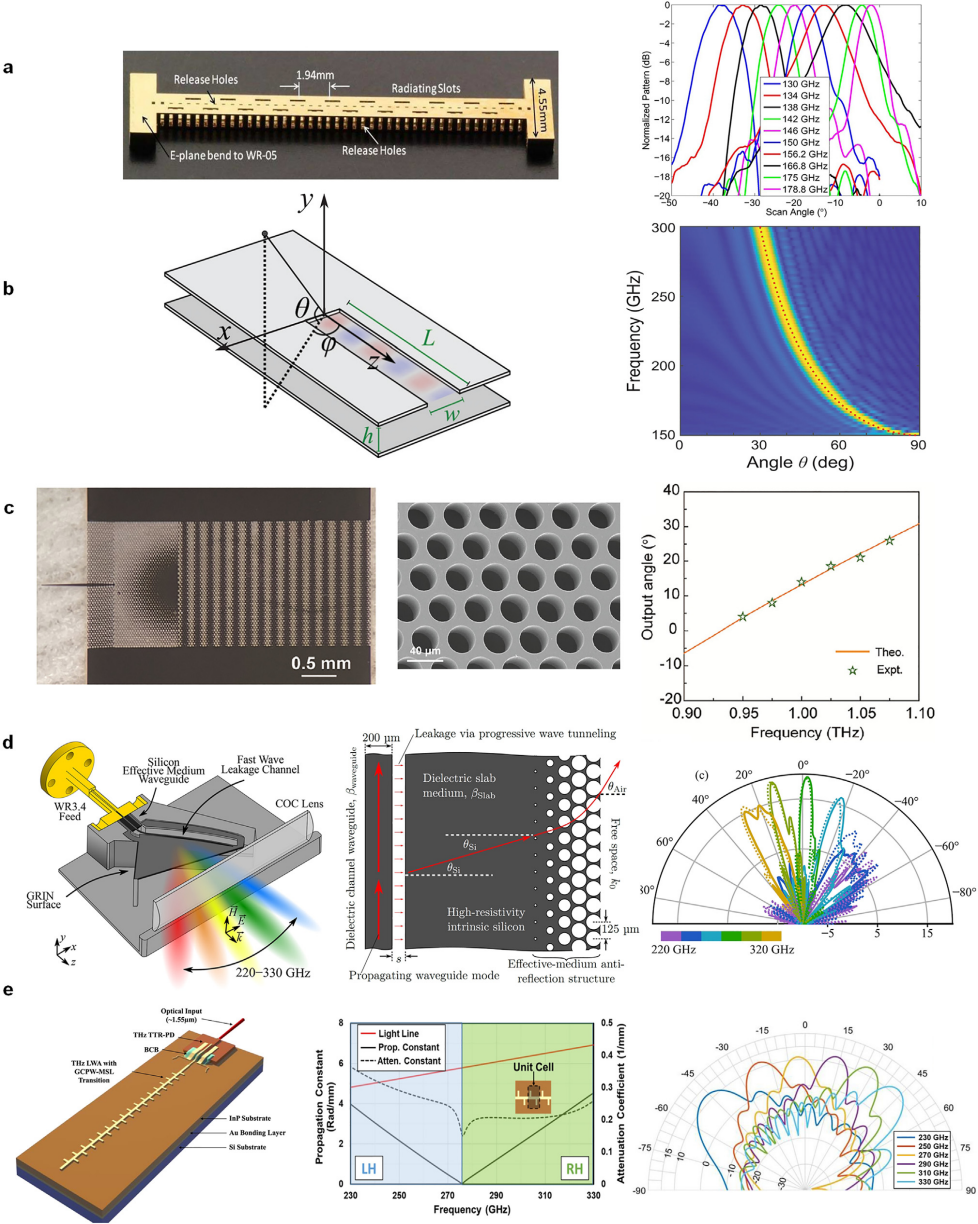
**THz LWAs using different classes of low-loss waveguiding structures. (**a) Hollow RWG with staggered longitudinal slots [84]. (b) TE_1_ mode PPWG with a longitudinal long slit [93]. (c) Silicon substrate with periodic air hole. [97]. (d) Dielectric waveguide leaking power to the neighboring dielectric slab [98]. (e) Integrated microstrip combline LWA based on indium phosphide (InP) wafer [101].

The construction of LWAs relies on waveguiding structures, along which traveling waves propagate while simultaneously leaking power to the neighboring medium (e.g., free space) through continuously or discretely distributed radiating discontinuities [83]. It is a well-recognized fact that various kinds of LWAs would be devised after the invention of new waveguiding structures, which can be justified by retrospectively viewing the past research activities that have been conducted since the last century to date in microwaves. It can be seen that a great variety of non-planar and planar waveguiding technologies such as rectangular waveguides (RWGs), microstrip lines, substrate-integrated waveguides, spoof surface plasmonic polaritons, and gap waveguides have been invented one after another and then deployed for constructing multifarious LWAs [83]. While the theories of LWAs show no difference in whether the working frequency is in microwaves or THz waves, special considerations should be imposed on the section of suitable waveguiding structures for developing THz LWAs, especially focusing on transmission losses and fabrication issues. Typically, considering that conventional dielectric materials may become very lossy as frequency increases to THz, an advisable way is to use hollow or air-filled waveguiding structures such as the metallic RWG [[84], [85], [86], [87], [88], [89], [90], [91]] and PPWG [[92], [93], [94], [95]] to implement LWAs. Fig. 4a depicts a 130–180 GHz longitudinally slotted hollow RWG LWA, in which a micro-fabrication technique based on a sequential copper deposition process was used for prototyping [84]. Although no dielectric loss is presented, the conductor loss still exists in the hollow RWG and generally manifests an increasing trend with frequency [122]. Not to mention the surface roughness introduced by the fabrication process may also deteriorate the conductor loss. To reduce the conductor loss of a hollow RWG, we may simply remove its top and bottom broad walls since they contribute most of the conductor loss (assume only the dominant TE_10_ mode propagates in the RWG) [122]. Such a modified waveguide corresponds to a TE_1_ mode PPWG, which manifests a decreasing conductor loss with frequency due to the electrical lines of the TE_1_ mode being parallel to the two constituent metal plates. This means that the TE_1_-mode-based PPWG is a promising candidate to work as THz waveguiding structures [123,124] and to develop THz LWAs [[92], [93], [94], [95]], as can be found in Fig. 4b where a longitudinal slit working as a continuously distributed radiating discontinuity is cut on the upper plate of the PPWG to trigger power leakage and constitute the antenna aperture [93]. It is interesting to recall that on the way to exploiting millimeter waves in the last century, great efforts have been made to cope with the conductor loss of waveguiding structures. Several milestones of waveguiding structures such as H-guide [125], Groove-guide [126], and nonradiative dielectric waveguide (NRD) [127] were consecutively invented at that time, and they all share the similar principle of low conductor loss as that of the TE_1_ mode PPWG.

While the hollow RWG and PPWG are promising for developing highly efficient THz LWAs, the dielectric waveguide is also a good low-loss waveguiding technique without suffering from the conductor loss mechanism [96,122]. Fig. 4c shows a superheterodyne-inspired LWA antenna based on the high-resistivity silicon (Si) substrate operating at 1 THz [97]. The spatial modulation of the Si waveguide through etching air holes enables the −1st-order space-harmonic to become fast and radiate into free space with a frequency-dependent beam. Instead of introducing periodic scatters on dielectric waveguides (e.g., grooves, metal strips, and air holes) to evoke space-harmonics-based leaky-wave radiation [81,83], Fig. 4d depicts a uniform dielectric waveguide that is made leaky by adjacently placing a dielectric slab [98]. While the phase velocity of the modal fields propagating in the dielectric waveguide is faster than that of the neighboring dielectric slab, the leaky-wave phenomenon can be triggered between the two waveguides while presenting a frequency-dependent angle of power leakage. The propagated waves in the dielectric slab are then refracted to the free space to form a frequency-scanned beam.

Compared to the waveguiding structures discussed above, the microstrip line does not present comparably good transmission performances at THz waves because of the unavoidable radiation loss apart from the dielectric and conductor losses. Nevertheless, the salient benefit of using a microstrip line at THz is its convenience of integration with other active and passive components using the same semiconductor platforms [[99], [100], [101], [102], [103], [104]]. As shown in Fig. 4e, an integrated microstrip combline LWA was proposed using the InP wafer [101], which is dedicated to the full integration with InP photodiodes and thus paves the way for realizing monolithically integrated beam-scanning THz transmitter chips.

### 4.2 Diffraction gratings—the type using spatial-wave illumination

While the transmission losses involved in waveguiding structures may become significant in the THz band, it would be somewhat difficult for a THz LWA to have a long radiating aperture of typical many wavelengths considering that the guided waves or leaky modes may attenuate quickly due to material losses and power leakage. In this case, not only the radiation efficiency of THz LWAs would suffer, but a high-gain beam, which is necessary to compensate for the large propagation loss of THz waves in wireless transmission, may not be easily generated. In this case, diffraction gratings may take over the job of LWAs to produce a frequency-scanned high-gain beam at the THz band [[106], [107], [108], [109], [110], [111], [112], [113], [114], [115], [116], [117], [118], [119], [120], [121]], which is simply because the spatial-wave illumination or feeding nature of diffraction gratings prevents them from encountering the material losses that are embedded essentially in LWAs. This subsection will discuss three different approaches to designing diffraction gratings with a frequency-scanned beam.

**Fig. 5:**
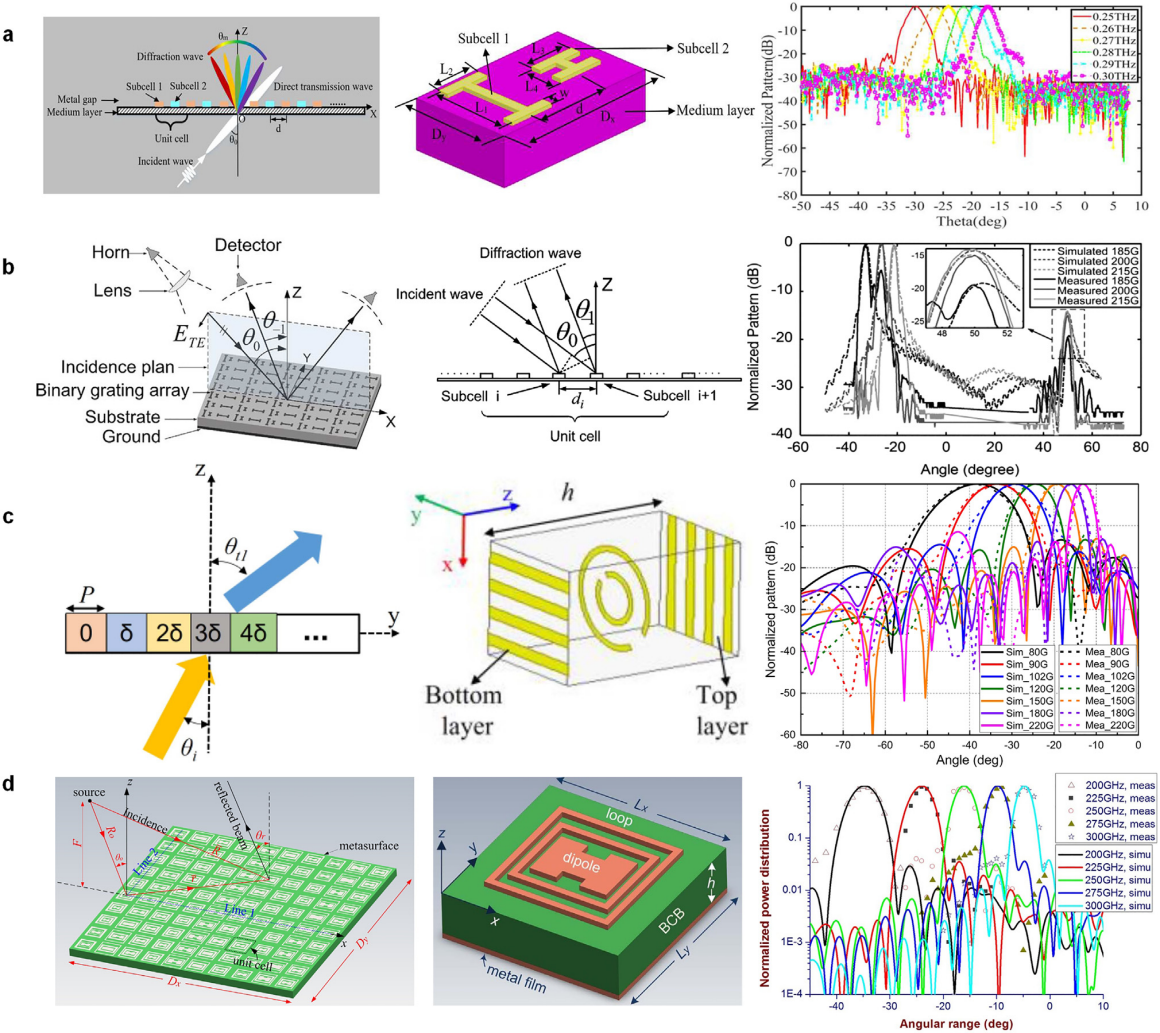
**Different kinds of THz diffraction gratings with frequency beam-scanning behavior**. (a) Transmissive type based on −1st-order diffraction beam [110]. (b) Reflective type using −1st-order diffraction beam [111]. (c) Transmissive type based on the 0th-order diffraction beam and the frequency-independently constant phase gradient [117]. (d) Reflective type based on the 0th-order diffraction beam and the multi-frequency phase-matching approach [118].

The first approach is related to the use of a higher-order diffraction beam (which has the same physical meaning as the space-harmonic-related beam in quasi-uniform and periodic LWAs [81]) while suppressing the fundamental one in the visible region [[106], [107], [108], [109], [110], [111], [112], [113], [114], [115]]. For a general diffraction grating, regardless of the transmissive or reflective type, the 0th-order diffraction beam is usually in the visible region and has a fixed beam direction regardless of frequency. Comparatively, all the higher-order beams are dispersive and behave in the manner of moving closer to the 0th-order one as frequency increases, and most of these beams are spatially invisible in general cases depending on the incidence angle, frequency, and unit-cell period. Considering that an individual frequency-scanned beam (e.g., −1st-order beam) in the visible region is usually required just as what LWAs generally manifest, the 0th-order beam of diffraction gratings should be effectively suppressed. To this end, we may design a grating unit cell consisting of several different substructures or sub-cells, whose phase gradients and distances are specially engineered to ensure that the radiation of these sub-cells can have a coherent superposition towards −1st-order beam direction. The involved mechanism can be easily understood according to the antenna array theory [9]. Specifically, the entire unit cell can be seen as a composite antenna element or subarray whose maximum radiation is aligned to the direction of the −1st-order beam while presenting low directivity to other directions (including the direction of the 0th-order beam). Consequently, the 0th-order beam will be suppressed by such an engineered “element pattern” and most of the incidence power is transferred into the desired −1st-order beam (provided that no other higher-order beams appear in the visible region). Figs. 5a and b respectively depicted the transmissive and reflective type THz diffraction gratings that radiate a frequency-scanned −1st-order beam in the visible region [110,111]. Their fundamental 0th-order beams are effectively suppressed by specially designing the unit cells following the abovementioned coherent superposition principle of sub-cells.

It is interesting to mention that the diffraction gratings discussed above resemble periodic-type LWAs in that they all use higher-order beams (i.e., the −1st-order) to form a frequency-scanned beam [[81], [82], [83]]. However, one of the significant differences between the two is that the 0th-order beam of periodic LWAs is in the invisible region because of the slow-wave nature of the relevant guided waves (no efforts are required to suppress the 0th-order beam of periodic LWAs). Recall that the quasi-uniform-type LWAs are characterized by a sub-wavelength periodicity and use the 0th-order space harmonic for radiation (the relevant higher-order modes are generally invisible over the frequency band of interest). This inspires us to deduce whether the diffraction gratings can be made following such a manner if possible. Indeed, when using a subwavelength periodicity for diffraction gratings, all the higher-order beams will be pushed into the invisible region and only the fundamental one is left for radiation. While the direction of the 0th-order beam is not changed with frequency that sharply contrasts with that of quasi-uniform LWAs, some measures should be adopted to make it scannable with frequency. One feasible way is to introduce phase gradients between the two adjacent unit cells (i.e., the second design approach), which evolves from the generalized Snell's law and usually can be found in the design of reflect-/transmit-arrays or metasurfaces. Specifically, when the phase gradient is not changed with frequency or is not a linear function of frequency, the relevant 0th-order beam will be scanned with frequency [116,117]. Fig. 5c depicts an example following this mechanism [117]. On the other hand, as shown in Fig. 5d, we can also adopt an approach called “multi-frequency phase matching” for the same purpose of generating a frequency-scanned 0th-order beam [118]. Different from the design of conventional reflect-/transmit-arrays or metasurfaces in which only a single angle of collimation is paired at a certain frequency point, the “multi-frequency phase matching” approach simultaneously focuses several consecutive design frequencies and each of them has its corresponding angle of collimation [[118], [119], [120], [121]]. In other words, this approach can be similarly viewed as evolving from conventional reflect-/transmit-arrays or metasurfaces by extending the design specifications of the paired “frequency” and “angle of collimation” from one to multiple, which constitutes the third approach to design diffraction gratings with a frequency-scanned beam. By carefully optimizing the dimensions of each unit cell to render it simultaneously meet the phase profile requirements at multiple frequencies/angles, the designed gratings can produce different beam angles at different frequencies, i.e., the frequency beam-scanning behavior. It is interesting to mention that this “multi-frequency phase matching” approach also adopts the 0th-order space harmonic for radiation purposes.

## 5 Reconfigurable metasurfaces

Metasurfaces, consisting of specifically designed subwavelength-scale elements, represent the two-dimensional equivalents of metamaterials [[128], [129], [130], [131], [132], [133], [134], [135], [136], [137], [138], [139], [140]]. Metasurfaces enable enhanced wave-matter interaction within ultra-thin structures and can flexibly manipulate the properties of incident electromagnetic waves, such as amplitude, phase, polarization, direction, etc. [[141], [142], [143], [144], [145]]. By incorporating active components or tunable materials, metasurfaces can dynamically change their surface impedance upon external stimuli and enable post-fabrication wavefront control. At microwave frequencies, discrete diode-based components, such as PIN diodes and varactor diodes, are commonly adopted for developing tunable or reconfigurable metasurfaces. However, these components/materials suffer from prohibitively high loss when the working frequency increases to the THz band, not to mention that their footprints may not be scaled and adapted to the short wavelength of THz waves. Researchers have thus turned their attention to other sophisticated approaches that match the characteristics of THz waves, such as using integrated CMOS/HEMT semiconductor components, phase transition materials [e.g., VO_2_, Ge_2_Sb_2_Te_5_, GeTe], LC, graphene, and MEMS technologies. Among these, CMOS/HEMT semiconductor transistors can achieve fast switching speeds up to 5 GHz but generally operate in the lower THz frequency band (around 0.3 THz). In contrast, phase change materials, LC, graphene, and MEMS can operate at higher THz frequency bands but typically exhibit a lower response speed on the order of several hertz to kilohertz, depending on the adopted stimuli methods and specific geometrical structures of metasurfaces.

**Fig. 6:**
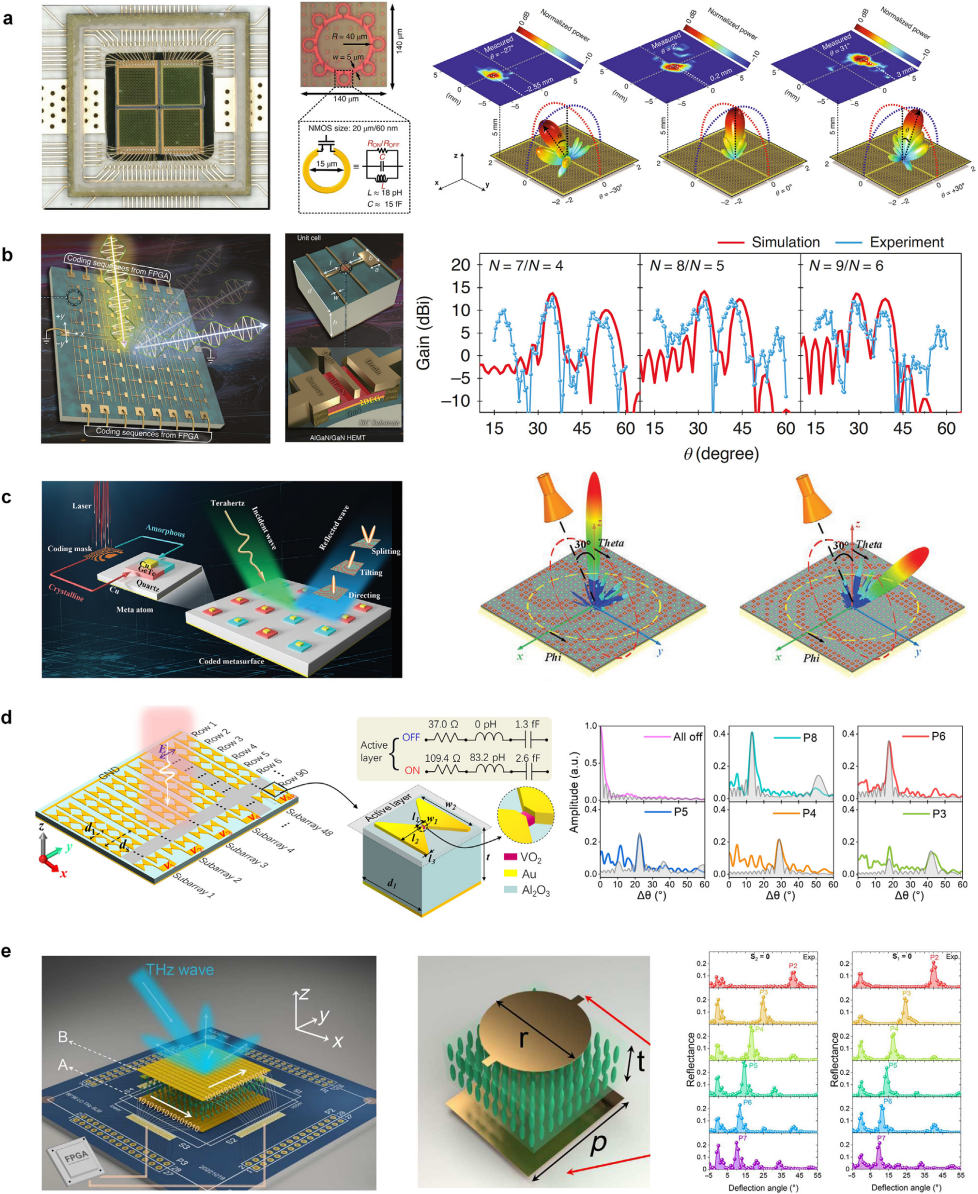
**THz reconfigurable metasurfaces integrating with different active materials for beam scanning**. (a) A CMOS-based high-speed programmable metasurface chip for dynamic beamforming and holographic imaging [128]. (b) A GaN HEMT-based programmable metasurface for multifunctional wavefront engineering [129]. (c) A GeTe-based 1-bit coding metasurface for THz beam scanning [130]. (d) A VO_2_-based electrically addressable reflective metasurface for self-adaptively THz beam deflection [131]. (e) An LC-based metasurface with a crossbar control network for 2-D beam scanning [132].

Venkatesh *et al*. reported a 2 × 2 tiled 0.3 THz transmissive metasurface chip based on the 65 nm industry-standard CMOS technology, as shown in Fig. 6a [128]. The metasurface consists of 24×24 loop-shaped meta-atoms, each incorporating 8 CMOS transistor switches to implement different C-shaped split-ring resonators. In addition to realizing a transmission amplitude with a modulation depth of 25 dB and a transmission phase range of 260° at 0.3 THz, the reported reconfigurable CMOS metasurface in [128] can also steer the beam from −30° to +30° and be used for multi-beam generation and holographic imaging applications. Additionally, a 1-bit, 98×98-element reflectarray based on 22 nm CMOS technology has been reported to generate a 1° beamwidth and beam-scanning range of ±60° at 0.256 THz [146]. This THz beam-scanning reflectarray has been utilized to implement a THz FMCW radar for 3D sensing applications. Apart from the CMOS technology, GaN HEMTs can offer nanosecond-level response speeds and are another promising semiconductor device for THz reconfigurable control [147]. A GaN HEMT reflective metasurface with 1-bit phase modulation has been demonstrated to achieve a beam scanning range from 20° to 60° at 0.34 THz [129], as shown in Fig. 6b. There are also other HEMT-based THz metasurfaces reported in the open literature, including a spatial amplitude modulator at 0.34 THz [148], a spatial phase modulator at 0.352 THz [149], and a waveguide phase modulator at 0.26 THz [150].

Phase transition materials can switch between their two amorphous and crystalline states, which can serve as insulators and conductors, respectively, upon external thermal, electrical, or optical stimuli (or a combination of them [137,138]). The unique reversible binary states of phase transition materials open the door for reconfigurable THz metasurfaces through a meticulous design of meta-atom structures. For example, a hybrid metal and nonvolatile phase-change GeTe material meta-atom has been designed to achieve a 1-bit 0/π reflection phase difference at 0.3 THz, as shown in Fig. 6c [130]. By using laser irradiation of the metasurface aperture in a pre-designed pattern, the metasurface can be reconfigured to control beam tiling and splitting. In addition to the GeTe material, VO_2_ is another promising THz phase transition material that exhibits a tremendous conductivity change between its insulator and metal states. A thermally controlled VO_2_-based reconfigurable Janus metasurface has been demonstrated to achieve asymmetric transmission of THz waves and directional THz holography at 0.99 THz [151]. In another example [131], a VO_2_ microbridge is incorporated into the middle of a bowtie meta-atom to achieve a 180° reflection phase difference between the insulator and metal states, as shown in Fig. 6(d). This metasurface can realize 1-D beam scanning over 42.8° and self-adaptively adjust the beam deflection direction at 0.425 THz. Other VO_2_-based metasurface devices include an intensity modulator with a modulation depth of 97% over 0.67 THz [152], an amplitude and phase modulator with a modulation depth of 71% at 0.79 THz, and a 90° phase shift over a 70 GHz bandwidth [153], and a beam-steering metasurface with a 44° deflection angle range at 100 GHz [154].

LCs exhibit a strong birefringence effect and can generate tunable refractive indices with a driven electric field or voltage [155]. The LC-based spatial-fed phase shifters discussed in Section 3 can be adopted as the building blocks for developing beam-scanning reflective/transmissive metasurfaces. For example, a 1-bit transmissive metasurface based on a symmetry-breaking Fano resonator and LCs was reported in [156] to achieve 1-D beam scanning at 0.426 THz. A 1-bit reflective counterpart, with the LC embedded into the two metallic meta-atom layers, was developed for 1-D beam steering at 0.672 THz [157]. An LC-based reflectarray with a 330° tunable reflection phase range was demonstrated to achieve a 1-D beam scan over 55° from 96 to 104 GHz [158]. Furthermore, as shown in Fig. 6(e), a crossbar structure through the modulo-addition of column and row coding matrices was proposed at 0.728 THz, with the functionality of 2-D beam scanning realized [132]. A 16×16 addressable transmissive metasurface was demonstrated to manipulate near-field THz wave at 103 GHz, including near-field printing imaging, 3-D energy convergence, and Bessel beam generation [159]. Notably, LC-based metasurfaces were demonstrated for use as spatial light modulation with a modulation depth of 75% at 3.67 THz [160] and reflective waveplates for polarization conversion [161]. In contrast to LCs, Graphene, a single layer of carbon atoms in a hexagonal arrangement, is another effective electrically tunable material for controlling THz waves due to its high-mobility charge carriers. A 1-bit graphene-based reflectarray has achieved a dynamic 1-D beam scan range of 25° by changing the period of the super unit cell at 0.977 THz [162]. Additionally, a 256-pixel graphene-based spatial light modulator with an electrically tunable transmission coefficient has been demonstrated for THz imaging applications [163].

MEMS technologies can also be used for reconfigurable THz metasurfaces with the benefits of low insertion loss and wide bandwidth. A spatial light modulator consisting of 768 actuatable MEMS mirrors has been reported to achieve an amplitude modulation contrast higher than 50% from 0.97 to 2.28 THz. Each pixel can be electromechanically forced into an inclined position of 35° to diffract the incident THz wave [164]. Furthermore, Fano resonances in a reconfigurable MEMS metasurface have been utilized to perform XOR, XNOR, and NAND logic operations of two controlled electrical input signals at 0.55 THz [165]. A similar approach using electric split-ring resonators as switchable meta-bits has been employed in [166] to construct a reconfigurable THz metamaterial with reconfigurable transmission output analogous to NOR and AND logic operations.

## 6 Conclusion and general remarks

While human footsteps regarding the exploitation of electromagnetic spectrum have never stopped since the famous experimentation about the generation and detection of electromagnetic waves conducted by Heinrich Hertz in 1887–1888 [167], research activities concerning closing the “THz gap” and pushing THz waves into practical use have attracted much more attention than their historical counterparts of exploiting the spectrum from RF to microwaves when looking retrospectively. Since THz waves, compared to microwaves, are subject to larger losses in wireless transmissions and should be thus equipped with a more directional antenna beam for practical applications, the significance of beam-scanning technologies in THz wireless has never reached such a height than it was in their counterparts of microwaves. To provide general readers with the basic knowledge of THz beam-scanning technologies, we have briefly reviewed in this article four distinct mechanisms available in the open literature, i.e., mechanical beam-scanning, phased arrays, frequency beam-scanning, and reconfigurable metasurfaces. While most of these beam-scanning schemes have existed for a long time in microwaves, more challenges are presented when adapting them to the THz band. For example, although performing beam-scanning with macroscopic and/or microscopic mechanical motions is the most straightforward method, the relevant scanning speed is usually limited and can hardly meet the ever-increasing requirements of future THz systems. Moreover, considering the very short wavelength of THz waves, it is not easy to assemble high-precision actuators (or servo systems) with the THz antenna structures to ensure sophisticated control of the beam direction. Compared to mechanical beam scanning, the phased array technique can provide precise and agile beam-scanning performance by properly phasing the array elements in either passive or active architectures. Based on the principles of how to generate THz waves, i.e., up-conversion from microwaves or down-conversion from optical waves, the relevant phase-shifting processes required for the phased beam-scanning can be performed in either microwave or optical domain (or in the THz band directly). Notably, from the perspective of full-electronics (especially the IC-based THz systems), the insertion losses of feeding networks and phase-shifting devices may impose great challenges for the large-scale deployment of THz phased arrays. In this sense, the emerging topological waveguiding technology based on valley photonic crystals [[168], [169], [170], [171]] may exhibit its great potential in high-performance THz beam-scanning with the benefits of low loss and compact features as well as the high compatibility with Si-based CMOS integration platforms. A typical example is the recently proposed THz on-chip topological beamformer [171] with phototunable multi-beams and 360° azimuthal coverage presented. On the other hand, it is seen that the significance of microwave photonics has increased in the THz era [3,13], simply because of its virtues of supporting the generation of higher-frequency THz waves and larger signal bandwidth as well as possessing lower transmission loss than the counterparts of full-electronics. Parallel to the rapid development of full-electronic integration technology, the field of microwave photonics has also significantly evolved and become more “integrated”. It is envisioned that integrated microwave photonics, which leverages integration platforms such as Si, InP, or lithium niobate (LN) (or uses a heterogeneous integration scheme) and combines several functional components like laser sources, electro-optic modulators, optical phase shifters, photodiodes, and antennas on a single chip will be a critical enabler for future high-performance THz beam-scanning wireless systems [3,13,[172], [173], [174]].

Frequency-enabled beam-scanning using guided-wave-based LWAs and spatial-wave-based diffraction gratings can steer the beam by simply varying the frequency and is thus free from using any mechanical or electrical/optical phase-shifting devices. We have reviewed several low-loss waveguiding structures that are suitable for constructing THz LWAs and also summarized three different mechanisms to design diffraction gratings. However, the beam-scanning performances of current THz LWAs and diffraction grating are generally limited, manifesting a narrow scanning range, slow scanning rate, unstable beam shape (beamwidth and gain), nonlinearity of the scanning function, etc. [83]. Addressing these technical issues is an important aspect of THz frequency beam-scanning when considering its practical applications [[3], [4], [5], [6]]. While LWAs compared to diffraction gratings can be more easily integrated with other passive and active components, it is believed that LWAs may find more potential in compact, highly integrated, and cost-effective frequency-scanning THz wireless. In this sense, the conductor and dielectric losses of waveguiding structures should be well considered to develop THz LWAs, and new materials and advanced fabrication technologies as well as emerging waveguiding platforms can be envisioned for this purpose. Notably, the instantaneous bandwidth of a frequency-scanned beam is intrinsically limited [83], which somewhat makes this type of beam scanning lose its shine compared to mechanical and phased beam scanning in some THz applications.

Metasurface is an emerging enabling technology for THz beam scanning. Compared with other beam-scanning technologies, metasurfaces do not require complicated and lossy feeding networks and can easily integrate with tunable materials for agile beam management. By incorporating tunable components or materials (e.g., GeTe, VO_2_, LC, graphene, and MEMS) into the meta-atom structure, metasurfaces can be made reconfigurable, which can flexibly change the aperture's reflective or transmissive phase distribution for THz beam-scanning upon external individual or combined stimulus like electrical bias, thermal excitation, optical pumping, etc. However, the phase control in current reconfigurable metasurfaces is generally accompanied by amplitude modulation, and innovating meta-atom structures and co-optimizing them with active materials is important to implement independent amplitude and phase controls. Furthermore, a promising development direction for THz reconfigurable metasurfaces is the integration with signal detection, which leads to the development of THz intelligent metasurfaces (as an upgraded version of conventional reconfigurable metasurfaces). For example, a THz intelligent metasurface proposed in [131] can adaptively adjust the beam direction according to the detected THz signal for eliminating coverage dead zones and stabilizing the reflected THz power. Another promising direction we can follow is to incorporate temporal dimension into the design of metasurfaces to develop the so-called spatiotemporally modulated or space-time-coding metasurfaces (STCM) [141,142,175,176]. For example, an interesting STCM with 1-bit amplitude modulation has recently been reported in [141], which can manipulate all the fundamental properties of EM waves’ properties including amplitude, phase, frequency, momentum, and polarization, simply depending on spatiotemporally ON—OFF switching the meta-atoms. These exciting functionalities cannot be easily achieved by conventional metasurfaces that only leverage the modulation of spatial dimension, thereby demonstrating the benefits and capabilities of STCMs. While STCMs have been extensively studied at microwave frequencies for various applications such as Doppler cloaks [177], direction finding [178], analog signal processing [179], simplified transmitters [180], and real-time imaging [181], it is envisioned to they will also find similar potential in enabling premium THz wireless just as a natural evolution from microwaves. For this purpose, several technical challenges like high-speed switching mechanisms and low-loss control circuits should be well considered for developing THz STCMs, and relevant implementation schemes or solutions can be inspired and adapted from those integrated chip-level THz reconfigurable metasurfaces [[128], [129], [130], [131], [132]].

While most of the THz beam-scanning technologies reviewed in this article can find their microwave counterparts that share similar fundamental principles, there also exist solutions that uniquely belong to the THz domain and they are generally related to the way of how generating THz waves. A typical example is related to PCAs in which optical pumps (ultrafast femtosecond lasers) and bias voltages are combined to enable THz generation; the required beam-scanning can be then performed and explained following the mature antenna theory in microwaves [76,[78], [79], [80]]. In summary, the past two decades have witnessed remarkable progress in the development of THz beam-scanning technologies, which can further reduce the “THz gap” and push forward the exploitation of THz waves. Nevertheless, there remains a long distance for THz waves to have comparable technical maturity as microwaves. Efforts for this purpose necessitate interdisciplinary collaboration involving antenna technology, material science, mechanical engineering, metasurfaces, etc. By leveraging interdisciplinary expertise and fostering innovation across multiple domains, high-performance THz beam-scanning antenna technologies are anticipated in the short future, which will certainly reshape the landscape of next-generation wireless systems such as communication, sensing, and imaging.

## Declaration of competing interest

The authors declare that they have no conflicts of interest in this work.
